# Trophic Factors from Tissue Stem Cells for Renal Regeneration

**DOI:** 10.1155/2015/537204

**Published:** 2015-05-18

**Authors:** Kenji Tsuji, Shinji Kitamura

**Affiliations:** Department of Medicine and Clinical Science, Okayama University Graduate School of Medicine, Dentistry and Pharmaceutical Sciences, 2-5-2 Shikata-cho, Okayama, Okayama 700-8558, Japan

## Abstract

Stem cell therapies against renal injury have been advancing. The many trials for renal regeneration are reported to be effective in many kinds of renal injury models. Regarding the therapeutic mechanism, it is believed that stem cells contribute to make regeneration via not only direct stem cell differentiation in the injured space but also indirect effect via secreted factors from stem cells. Direct differentiation from stem cells to renal composed cells has been reported. They differentiate to renal composed cells and make functions. However, regarding renal regeneration, stem cells are discussed to secrete many kinds of growth factors, cytokines, and chemokines in paracrine or autocrine manner, which protect against renal injury, too. In addition, it is reported that stem cells have the ability to communicate with nearby cells via microvesicle-related RNA and proteins. Taken together from many reports, many secreted factors from stem cells were needed for renal regeneration orchestrally with harmony. In this review, we focused on the effects and insights of stem cells and regenerative factors from stem cells.

## 1. Introduction

Renal failure is one of the major healthcare issues. Although medical science has rapidly advanced, there are few effective treatments to rescue kidney injury or to activate kidney regeneration. Recently, stem cell therapies have been proposed for the regeneration toward different kinds of organ failures, such as cardiovascular diseases [1], neurodegenerative diseases [2], and kidney diseases [3]. As to the kidney, many stem cell therapies are reported to be effective in variety of kidney injury models and the therapies are expected to be useful treatment against kidney injury [4].

Nevertheless, the mechanisms of the stem cell-induced regeneration are still controversial. Among these mechanisms, direct differentiation of stem/progenitor cells into renal mature cells have been reported as the direct therapeutic mechanism [5]. Recently, many reports revealed that trophic factors from stem cells might be the important contributor for kidney regeneration [6, 7]. In these reports, there are different kinds of stem cell sources, such as bone marrow-derived mesenchymal stem cells, adipose-derived mesenchymal stem cells, and adult kidney stem/progenitor cells. These secreted factors are reported to regulate the cell proliferation, cell migration, cell differentiation, and immune systems as well as cell-cell interactions and circumstances around the injured space [8].

Among kidney injury, acute kidney injury (AKI) is happening in 2–5% of the hospitalized patients [9]. AKI results from different kinds of factors, such as toxin and renal ischemia [10]. Some cases of AKI are reversible and the recovery of the injured tubules needs the replacement of injured tubular epithelial cells [11]. The cell sources for the replacement of injured tubules have been controversial. Recently, Humphreys et al. reported that most predominant mechanism of repair after ischemic tubular injury is the surviving tubular epithelial cells [12]. In addition, Kusaba et al. reported that fully differentiated kidney epithelial cells repair injured proximal tubule [13]. They suggested that surviving terminally differentiated tubular epithelial cells dedifferentiate, proliferate, migrate, and replace the injured tubules [13]. These reports implied that stem cells such as mesenchymal stem cells (MSC) and adult kidney stem/progenitor cells are not the predominant cell sources for the replacement of injured tubules. And these reports highlighted the possibility that the role of the stem cells for regeneration might result from the indirect mechanism via secreted factors from these cells. These factors might activate the regenerative process, for example, via regulation of the cell proliferation, tubular cell dedifferentiation, cell migration, and circumstance condition such as inflammation. Actually, stem cells have been reported to secrete a variety of trophic factors and activate the regeneration [14]. Although detail mechanisms are still uncertain, it might open the new strategy to treat AKI or other kidney injury if we can elucidate the detail mechanism of indirect therapeutic mechanism via trophic factors from stem cells and translate into clinical medication treatment.

In this review article, we focused on the recent advances of the therapies related to the trophic factors from these stem cells.

## 2. Mesenchymal Stem Cells

MSCs can be established from adult tissues including bone marrow, adipose tissue, synovial tissue, liver, lung, umbilical cord, placenta, amniotic fluid, and connective tissues [15]. MSCs are the undifferentiated adult cells and have the ability to differentiate into mesenchymal-derived tissues, such as bone, muscle, fat, and other connective tissues [15]. MSCs also have the ability to migrate to damaged and injured space and secrete many kinds of factors which can influence cell characters and change circumstances, which result in tissue repair and regeneration.

MSCs implantation has been reported to be protective for many kinds of kidney injury models, including not only AKI models such as ischemia/reperfusion kidney injury models, cisplatin injury model, glycerol injury model, and mesangioproliferative glomerulonephritis model [16–19] but also CKD models such as 5/6 nephrectomy model, type 1 diabetes model, and unilateral ureteral obstruction (UUO) model [20–22].

Regarding indirect replacement into injured tubules, trophic factors from MSCs have been reported to be protective against kidney injury. Human adipose-derived MSCs inhibited podocyte apoptosis and injury by high glucose mainly via trophic factors [23]. Bone marrow derived MSCs protected against ischemic acute kidney injury via trophic factors [6]. Bone marrow derived MSCs protected the kidney from toxic injury by trophic factors which limited apoptosis and enhanced proliferation of the endogenous tubular cells [7]. These reports indicate that there are variety kinds of factors and therapeutic mechanisms via trophic factors. Stem cells, like MSCs, might activate renal residual cells for regeneration among many kinds of renal diseases. Rota et al. reported that human amniotic fluid stem cells improved cisplatin-induced kidney injury and the effect was mediated through a local paracrine including interleukin-6 (IL-6), VEGF, SDF-1, and IGF-1 [24]. Since different kinds of stem cells secrete specific and different concentrated factors, it seems to be important to elucidate each stem cells contribution for regeneration. And these reports also suggest that cell transplantation of stem cells themselves is not necessary and stem cells do have indirect therapeutic mechanism via secreted factors.

On the other hand, there are some reports which focus on the culture supernatant from stem cells. It is reported with using co-culture system that anti-inflammatory factor HGF and anti-fibrotic factor TSG-6 from MSCs ameliorate albumin-induced tubular inflammation and fibrosis [25]. Low serum cultured adipose tissue derived stromal cells ameliorated AKI and the effect was mainly induced by HGF from these cells [26]. In addition, Human embryonic MSC-derived conditioned medium can rescue kidney function in 5/6 nephrectomy-induced CKD model [27]. These reports focus on the importance of the combination of variety kinds of trophic factors to get more efficient therapeutic effects. Each factor from MSCs is effective for renal regeneration. However, the secreted factors from MSCs are different and the working effect for renal disease is different, too. Therefore, it is supposed that there is the possibility that culture supernatant from stem cells might be optimal condition for renal regeneration because the trophic factors from MSCs might be secreted orchestrally.

**Figure 1:**
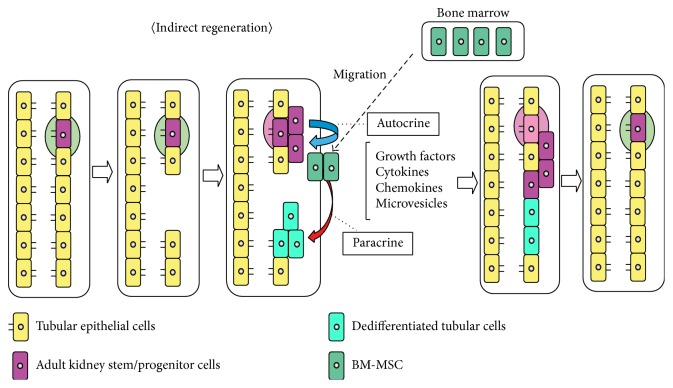
Shema of indirect therapeutic mechanism of stem cells against AKI. Not only the cell proliferation of stem/progenitor cells but also indirect factors such as growth factors, cytokines, chemokines, microvesicles et al. ameliorate injuryed tubular cells.

MVs from MSCs have been reported to be renoprotective. Human MVs derived from human MSCs can stimulate cell proliferation and inhibit cell apoptosis in vitro and activate recovery of glycerol-induced AKI mice model in vivo [28]. These effects were reversed with RNase treatment, suggesting MVs shuttled mRNA is the main contributor for renoprotection. Interestingly, they implied in the report that MVs from stem cells have the potential to induce dedifferentiation of mature cells which contribute to the tissue repair against injury. In addition, it is reported that human adult MSCs-derived MVs can protect against not only ischemia-reperfusion- (I/R-) induced AKI but also CKD [29]. These factors may open the new strategy to treat kidney injury. Taken together, MSCs contribute not only direct differentiation to kidney but also indirect trophic factors supply (Figure 1).

## 3. Adult Kidney Stem/Progenitor Cells

Adult kidney stem cells have been identified from some research groups in different kinds of technologies [4] (Table 1). These groups also reported that administration of the adult kidney stem cells contributed to renal regeneration. These reports especially focused on the direct therapeutic mechanism via replacement into injured tubules and there are few reports indicating the indirect therapeutic effects via secreted factors from these cells.

Hoechst 33342 was originally used for the isolation of HSC. In the same way, SP cells have been isolated from other organs including kidney [30, 31]. These cells have multilineage capacity and the ability to activate renal regeneration [31]. SP cell administration into kidney injury models, such as cisplatin-induced AKI, adriamycin nephropathy, and CKD models, revealed to be effective for kidney regeneration. In addition, these effects seemed to be mainly induced via paracrine effects from SP cells because there are few cell infusions into injured place [32].

Other adult kidney stem cells were isolated with the maker, Sca1^+^ cells [33]. When they injected these cells directly into the renal parenchyma, they differentiated into tubular phenotype and could contribute to kidney regeneration [33]. Other group isolated the kidney-derived stem cell line which was named multipotent renal progenitor cells (MRPC) [34]. They isolated MRPC using specific cell culture conditions which were similar to those used for the culture of bone marrow derived multipotent adult progenitor cells. These cells had spindle-shaped morphology and have self-renewal ability [34]. They also have the ability to differentiate into cells of all three germ cell layers. When they injected these cells into kidney injury models, they differentiated into renal tubules [34]. Other group reported adult kidney stem/progenitor-like cells by detecting slow cycling cells which represent the stem cell character using DNA labeling with BrdU [35]. They named the cells slow-cycling label-retaining cells (LRCs). LRCs were identified in renal tubular cells, papilla, and renal capsules. Interestingly, all tubular cells have the potential to become LRCs and proliferate after kidney injury, suggesting that differentiated tubular cells could dedifferentiate and have more immature characters when kidney injury occurs. The result suggested that LRCs were regulated via secreted factors at least to some extent. Other group was reported that CD 133^+^CD24^+^ adult kidney stem cells locate in the Bowman's capsule [36] and they were recently identified in the tubular compartment [37]. They both were positive for CD133, CD24, and Pax2, but there are some differences between these cells with respect to therapeutic effects. Tubular adult renal stem/progenitor cells (ARPCs) were reported to promote proliferation of surviving tubular cells and inhibited cisplatin-induced apoptosis [38]. These regenerative effects resulted from the ARCs-secreted inhibin-A and decorin mRNA [38]. Interestingly, this protective mRNA was shuttled by MVs. And the transcriptions from MVs differed from those from MSCs. These results suggested that there may be different roles via secreted factors between adult kidney stem cells and MSCs. Bussolati et al. also detected progenitor cells in the inner medullary papilla region [39] and they indicated that CD133^+^ cells from renal medulla possess higher differentiate ability and stemcellness marker compared to CD133^+^ cells from proximal tubules. When they evaluated the injection of CD133^+^ renal cells from human inner medulla in a model of glyceol-induced acute tubular injury model, they found the recovery of renal function and these effects were mediated via prevention of tubular cell necrosis and stimulation of resident cell proliferation and survival which is similar to MSCs [40]. Interestingly, they compared the therapeutic effects to MSCs and revealed that renal progenitor cells showed a high renal localization. Moreover, they also revealed the differences of secreted factors between renal progenitor cells and MSCs. Renal progenitor cells showed higher expression of plated-derived growth factor (PDGF), bFGF, leukemia inhibitory factor, and tumor necrosis factor *α* compared to MSCs. Taken together, kidney stem cells and MSCs secrete different kinds of trophic factors, which activate renal regeneration together. At the same time, there are important and significant differences between CD133^+^ renal progenitor cells from different segments, Bowman's capsule, S3 segment of proximal tubules, and inner medullary papilla region. These differences might be occurring from the different niche and environment of these cells. They might have the different roles about renal regeneration and the effects should be induced via trophic factors from these cells, at least to some extent.

Our group previously established an adult kidney stem/progenitor cell line (KS cells) from adult rat kidneys [41]. We isolated nephrons and separated into segments and cultured. We could isolate highly proliferative cells from single cell of S3 segment. KS cells showed cobblestone appearance and expressed not only mature tubular markers, such as aquaporin(AQP)-1, 2 and NaCl transporter (NaCl-tr), but also immature cell markers which are related to kidney development, such as paired box-2 (Pax-2), WT-1, and glial cell line-derived neurotrophic factor (GDNF). In vitro, KS cells have the self-renewal ability and can differentiate into mature tubular cells defined by AQP-1, 2 expressions. When we injected KS cells into AKI models, KS cells could differentiate and replaced to renal composed cells in injured tubules [41]. We also suggested the possibility that KS cells could contribute to regeneration by indirect mechanism via secreted factors from KS cells [42].

## 4. Trophic Factors for Renal Regeneration

Regarding trophic factors from MSCs, it is reported to secrete many kinds of factors such as HGF, EGF, IGF-1, FGF2, VEGF, BMP-7, TGF-b1, IL-6, IL-10, PGE2, granulocyte-colony stimulating factor (G-CSF), and macrophage-colony stimulating factor (M-CSF) [6, 8, 43, 44]. MSC-derived BMP-7 ameliorates diabetic glomerular fibrosis by inhibiting TGF-b signaling [45]. MSCs ameliorate podocyte injury and proteinuria in a type 1 diabetic nephropathy rat model via BMP-7 from MSCs [46]. HGF from MSCs ameliorated DN by inhibiting monocyte chemotactic protein-1 (MCP-1) expression [47]. Since MSCs have been reported to activate regeneration for different kinds of organs and MSCs migrate mainly from bone marrow after renal injury, MSCs should be one of the most important stem cells for renal regeneration.

Trophic factors such as growth factors, cytokines, and chemokines play important roles for many biological functions such as cell growth, migration, differentiation, apoptosis, inflammation, division, and signaling [8] (Table 2). Recently, the use of trophic factors has been advocated to stimulate kidney regeneration [48–50]. Growth factors injections against kidney injury have been reported. Miller et al. first reported that hepatocyte growth factor (HGF) administration against acute ischemia renal injury stimulated the recovery of kidney function and regeneration of proximal tubular epithelium [49]. In addition, they reported that EGF-like growth factor (EGF) or insulin-like growth factor-1 (IGF-1) administration against acute ischemia renal injury activated renal regeneration [49]. It is well known that HGF stimulates the cell proliferation of many kinds of epithelial cells and inhibits inflammation and fibrosis via cognate receptor HGFR/c-Met [50]. IGF-1 and EGF are known to be mitogenic for renal proximal tubules. Another factor, basic fibroblast growth factor (bFGF), is expressed in response to kidney injury [51]. Recent report revealed that bFGF can reduce functional and structural damage in chronic kidney disease (CKD) rat model [52]. bFGF inhibits apoptosis, activates epithelial condensation, and regulates Wilms' tumor 1 (WT-1) synthesis [53, 54]. In addition, FGF can activate cell proliferation of renal cells and regulate MSC differentiation [55]. Vascular endothelial growth factor (VEGF) is the important factor of the protective effect of MSCs using knockdown of VEGF by siRNA [16]. Human marrow-derived MSC-CM inhibited cisplatin-induced tubular epithelial cell death and these effects were reduced with anti-VEGF antibody [56], suggesting VEGF has the effect on kidney protection against toxic injury. VEGF regulates the survival and proliferation of different kinds of cells especially for endothelial cells and is also important for matrix remodeling, monocyte chemotaxis, and molecule expression related to adhesion. Bone morphogenic protein-7 (BMP7) inhibits transforming growth factor-b (TGF-b) induced fibrosis, reduces inflammation-related cytokines, and reduces cell apoptosis of epithelial tubular cells and podocyte whereas TGF-b and prostaglandin E2 (PGE2) suppress the inflammation by inhibiting lymphocyte activation [25, 26]. Recent report suggested that stroma cell-derived factor 1 (SDF-1) protects tubular epithelial cells against renal ischemia-reperfusion injury [57].

In addition to soluble factors such as growth factors, cytokines, and chemokines, microvesicles (MVs) were recently remarked as the way of cell-cell communication [58]. MVs are released from different kinds of cells [59]. MVs interact with nearby cells by mRNA, miRNA, proteins, transfer surface receptors, and surface ligands [59]. Recently, MVs shuttled RNA from stem cells have been reported to be renoprotective [39]. In addition, microRNAs are focused as new approach for kidney diseases. They are reported to have important roles for kidney development, disease, and regeneration [60]. The detail mechanism will be needed on the interaction between stem cells and micro RNAs for renal regeneration.

These trophic factors are reported to secrete from stem cells such as MSCs and adult kidney stem/progenitor cells. Taken together, trophic factors from stem cells should contribute to renal regeneration against injury and these effects might be induced by various factors orchestrally since stem cells secrete various kinds of factors. Stem cells might contribute to renal regeneration not only through direct differentiation into mature renal cells but also through the secretion of protective factors.

## 5. Conclusion

In this review paper, we focused that stem cells therapy contributes for regeneration via not only direct differentiation from stem cells to injured cells but also indirect regeneration with trophic factors from stem cell to residual cells. Though the detail movement is still explored, it is very important to resolve this mechanism. To elucidate the mechanism, stem cell therapy will be of clinical use. Now, there are few clinical medications for kidney injury. There are many trophic factors which can stimulate kidney regeneration, such as HGF, EGF, and IGF-1. There should be other unknown factors which can be renoprotective. Besides the soluble factors such as growth factors, microvesicle-related mediators including mRNA, miRNA, and proteins should be other therapeutic factors. These different kinds of trophic factors might contribute to regenerate kidney in orchestration with harmony from stem cells because the effects of each factor were different. It might be useful for culture supernatant of stem cells to regenerate renal injury because it can contain many unknown factors and might be adjusted as optical condition for regeneration. Although there are still much to be explored, we can open the new therapeutic method for kidney diseases when we could elucidate the detail mechanism about renoprotection related to trophic factors.

## Figures and Tables

**Table 1:** The summary of trophic effect of secreted factors from tissue stem cells.

Author	Cell line name	Species	Location
Hishikawa et al. [61]	SP cells	Mouse	Renal interstitial space
Challen et al. [62]	SP cells	Mouse	Proximal tubules
Inowa et al. [30]	SP cells	Human	Not identified
Dekel et al. [33]	Sca1^+^ cells	Mouse	Nontubular
Gupta et al. [34]	MRPCs	Rat	Renal tubules
Maeshima et al. [4, 35]	LRC cells	Rat	Renal tubular cells
Oliver et al. [63]	Slow-cycling cells	Mouse, rat	Renal papilla
Bussolati et al. [36, 39]	CD133^+^ CD24^+^ cells	Human	Bowman's capsule, inner medullary papilla, S3 segment of proximal tubules, interstitium
Sallustio et al. [37, 38]	CD133^+^ CD24^+^ cells	Human	Tubules, glomerulus
Sagrinati et al. [64]	CD133^+^ CD24^+^ cells	Human	Bowman's capsule
Lindgren et al. [65]	CD133^+^ CD24^+^ cells	Human	Renal proximal tubules
Kitamura et al. [41]	KS cells	Rat	S3 segment of proximal tubules

SP cells: Side population cells, MRPC cells: Multipotent renal progenitor cells, LRCs: Label-retaining cells, KS cells: kidney stem/progenitor cells.

**Table 2:** Summary of the adult kidney stem cells.

Factor	Trophic effect
HGF	Cell proliferation, anti-inflammation, antifibrosis
EGF	Mitogenesis (renal proximal tubules), anabolism
IGF-1	Mitogenesis (renal proximal tubules)
bFGF	Antiapoptosis, epithelial condensation, WT-1 upregulation, cell proliferation, MSC differentiation
VEGF	Cell proliferation, matrix remodeling, monocyte chemotaxis, adhesion protein upregulation
BMP-7	Antifibrosis, anti-inflammation, antiapoptosis
PGE2	Anti-inflammation
TGF-b	Anti-inflammation
MVs	Cell proliferation, antiapoptosis, dedifferentiation (renal mature cells)
microRNAs	Kidney development, homeostasis, and renal disease
